# Willingness to reduce alcohol consumption predicted by short-form video exposure, media involvement, psychological bias, and cognitive factor

**DOI:** 10.3389/fpsyg.2024.1213539

**Published:** 2024-01-30

**Authors:** Donghwa Chung, Yanfang Meng

**Affiliations:** ^1^School of Journalism and Communication, Central China Normal University, Wuhan, China; ^2^School of Journalism and Communication, Beijing Institute of Graphic Communication, Beijing, China

**Keywords:** short-form video exposure, short-form video involvement, reduced alcohol consumption, conscientiousness, theory of optimistic bias, survey

## Abstract

**Introduction:**

Through previous studies, Chinese college students are known to be prone to alcohol consumption, which can lead to health-risk behaviors such as high blood pressure, heart disease, stroke, liver disease, and digestive problems. However, little is known about how popular social media platforms (e.g., short-form video applications) can positively impact their willingness to reduce alcohol consumption. This study was guided by the theory of optimistic bias; we investigated the direct, mediated, and moderating effects of exposure to anti-alcohol consumption short-form videos and short-form video involvement on Chinese college students’ willingness to reduce their alcohol consumption.

**Methods:**

The current study has an empirical cross-sectional design and employed an online survey from September 1st, 2022, to November 1st, 2022. The survey specifically targeted Chinese college students, who are the most common users of short-form video applications. The accumulated data underwent rigorous examination, including hierarchical regression, mediation, and moderation analyses, all conducted using the PROCESS macro 4.0 within SPSS version 22.

**Results:**

A total of 434 participants, aged 18–24 years, were included in this study. There were mediating effects regarding Chinese college students’ exposure to anti-alcohol consumption short-form videos (β = 0.35, *p* < 0.01, 95% CI [0.17, 0.63]) and short-form video involvement (β = 0.44, *p* < 0.001, 95% CI [0.20, 0.65]) on their willingness to reduce alcohol consumption via reversed optimistic bias. Moreover, perceived prevention of heavy drinking control (β = 0.05, *p* < 0.001, 95% CI [0.01, 0.09]) played mediating roles in the relationship between exposure to anti-alcohol consumption short-form videos and willingness to reduce alcohol consumption.

**Conclusion:**

This study is one of the earliest studies to examine the intricate effects of exposure to anti-alcohol consumption short-form videos and short-form video involvement on the willingness to reduce alcohol consumption among Chinese college students. In addition, this study confirms that regardless of whether Chinese college students are conscientious, exposure to anti-alcohol consumption short-form videos did not increase their level of reversed optimistic bias. The empirical findings of this study are critical and can provide practical insights for Chinese health departments that encourage Chinese college students to minimize alcohol consumption.

## Introduction

Alcohol consumption is a term that refers to an individual’s act of orally ingesting a beverage containing ethanol, which is commonly studied in clinical and public health research (Collins et al., 2013; Zhou et al., 2016). Alcohol consumption has been associated with a list of diseases, including several cancers (e.g., hepatocellular carcinoma, esophageal cancer), glycogen depletion and liver cirrhosis (Bhavsar-Burke et al., 2021; He et al., 2021). Within the global population, college students are widely recognized as being particularly vulnerable to the risks associated with alcohol use. For instance, a recent study found that 59.78% of 1,382 students identified frequent drinking as a risk factor for injury (Moure-Rodríguez et al., 2014). In addition, this unhealthy behavior had a negative impact on their social lives and academic performance. Prior studies have identified heavy alcohol consumption as a significant factor leading to increased incidents of sexual harassment, poor academic performance, and subsequent dropout rates among college students (Abbey et al., 2004; El Ansari et al., 2020; Cimini and Martin, 2022). Previously, alcohol consumption had emerged as a major health issue in China, especially among the Chinese college student population (Guangming Daily, 2018). This has received significant attention from healthcare institutions and health education experts, who have called for the implementation of effective interventions. In response, numerous universities in China have developed a number of effective interventions. These interventions include the implementation of alcohol prohibition policies and guidelines for alcohol use and management, with the aim of significantly reducing alcohol consumption among Chinese college students (JYHYL, 2023). Recent evidence strongly indicates a dramatic decline in alcohol consumption and a significant decrease in willingness to purchase alcohol products among college students in China (Che, 2022; ZGHRZ, 2022). Such findings may have effectively increased college students’ willingness to reduce alcohol consumption (WRAC).

Previous cross-sectional studies have found that exposure to short-form video and social media involvement have increased individuals’ motivation to overcome alcohol consumption (Russell et al., 2021; Zeping, 2023). In addition, scholars have extensively examined key determinants such as attitudes toward alcohol (Morgenstern et al., 2011), self-control (Geurts et al., 2020), and perceived risk (Wolburg, 2001) that contribute to individuals’ willingness to reduce alcohol consumption (WRAC) across countries. The majority of studies have examined both direct and mediated effects of factors on WRAC under social psychological and cognitive theories (e.g., theory of planned behavior, theories of motivation, and social cognition) (Caudwell et al., 2019; Ajzen and Schmidt, 2020; Hagger and Hamilton, 2021). However, there remains a significant lack of studies on alcohol consumption that incorporate psychological bias theories, such as the optimistic bias, in the context of China. More research is warranted to explore how exposure to short-form videos or media involvement might help mitigate misperceptions of alcohol risk among Chinese college students, and whether a reverse optimistic bias (ROB) might influence their engagement in WRAC. These questions remain unconfirmed.

As discussed above, investigating the current effects of short-form video media on WRAC among Chinese college students (aged 18–24) is both timely and crucial. This study aims to provide valuable insights into the effects of exposure to anti-alcohol consumption short-form videos (EAACSFV) and short-form video involvement (SFVI) on WRAC among college students in the context of China. Therefore, the mediated mechanisms involving psychological biase and cognitive factors, such as ROB and perceived prevention of heavy drinking control (PPHDC) are considered. In particular, we aim to provide practical guidance and policy implications for college students to reduce their alcohol consumption.

## Literature review

### Theory of optimistic bias

Individuals tend to make comparative estimates of risk when encountering risky life events (Lerner and Keltner, 2001). Specifically, their optimistic bias arises when they compare the chances of risky life events occurring to themselves vs. others (Chambers et al., 2003). Scholars have discussed the definition of optimistic bias from different aspects. One such example is psychological bias, which is how people overly focus on the self when making comparative judgments (Chambers et al., 2003). This bias generally occurs when individuals perceive risk through a social comparison process, rather than through a simple form of belief (Lipkus and Klein, 2006). Wei et al. (2007) claimed that optimistic bias is people’s tendency to overestimate their likelihood of experiencing positive events and underestimate their likelihood of experiencing negative events in the future.

The theory of optimistic bias has been applied to examine individuals’ psychological biases toward risk within numerous research fields. Studies have shown that optimistic bias is one of the key factors that can predict individuals’ changes in perception and behavioral decisions (Hatfield and Job, 2001). For example, this has been seen in the context of environmental degradation (Hatfield and Job, 2001), engaging in COVID-19 prevention measures (Fragkaki et al., 2021), and preventive health behaviors (Chew et al., 2002). A recent study demonstrated that during the severe phase of the COVID-19 pandemic, citizens’ perceived risk was positively related to responses to risk. Ultimately, their assessment of risk was affected by optimistic bias and, as a result, this offset their level of engagement in preventive health behaviors (Lee et al., 2022). However, quite a few pieces of evidence indicate that there is also a negative impact of information exposure and media involvement on optimistic bias (Thompson et al., 2002; Van der Eijk et al., 2013). In the current study, ROB refers to individuals’ assumption that they are more vulnerable to the harmful consequences of alcohol consumption problems than they actually are. To the best of our knowledge, the academic literature has paid little attention to exploring whether ROB mediates the relationship between short-form video media effects (EAACSFV and SFVI) and preventing individuals’ health-risk behaviors (WRAC).

### Effects of EAACSFV and SFVI on WRAC, psychological biases, and cognitive factors

In China, short-form video applications have been recognized as one of the most popular communication applications and have largely attracted the interest of college students (Yang et al., 2021). According to the 2023 Douyin Health Science Data Report, there are a significant number of short-form health videos created by three different providers: certified doctors on Douyin, health institutes, and user-generated content. In particular, content created by certified doctors ranks among the highest, with an average of 35,000 health-related videos produced daily (Zhiniao, 2023). The latest studies have suggested that aside from delivering entertaining content to short-form video users, media platforms (e.g., Douyin) have also provided a large number of health information sources, which have increased individuals’ health awareness (Southwick et al., 2021) and health knowledge (Pudentiana Rr et al., 2022). Specifically, credible health-related short-form videos have induced individuals’ engagement in preventive health behaviors (Kong et al., 2021; Jaime et al., 2022). Furthermore, these applications have created a social communication environment where users can discuss and share health-related issues with others (Comp et al., 2021). In this study, EAACSFV is defined as the frequency at which individuals’ view anti-alcohol consumption contents on short-form video applications. Additionally, SFVI refers to engaging in interpersonal communication on short-form video applications (e.g., discussing alcohol consumption issues and sharing their opinions through commenting, liking, or sharing). A recent study has demonstrated that health information exposure is one of the key predictors of individuals’ healthy behaviors (Shen et al., 2018), such as engaging in physical activity (Viswanath et al., 2007), adopting behaviors to maintain a healthy weight (Rodgers, 2016), and being willing to get vaccinated (Zimand-Sheiner et al., 2021). A prior study also identified that social media involvement increases individuals’ intention to acquire healthy eating behaviors (Zhou and Krishnan, 2019). Similarly, Cao et al. (2017) indicated that the greater an individual’s engagement in social media, the more likely this activity will increase their HIV testing behavior. Moreover, Oeldorf-Hirsch et al. (2019) found that sharing tracked mobile health information online (social media involvement) reinforced a variety of health behaviors.

Previous studies have also revealed the relationship between social media effects (media exposure and media involvement) and ROB (Thompson et al., 2002; Kim and Hancock, 2015; van der Meer et al., 2023). A prior study indicated that college students who were exposed to HIV prevention videos experienced reduced perceptions of invulnerability (ROB) compared to other students (Thompson et al., 2002). In the same way, exposure to climate-change-related information has increased the level of college students’ first-person perception. That is to say, after being exposed to the information on social media, college students perceive the effects of environmental protection to be stronger on themselves compared to others (ROB) (Chung, 2019). Similarly, Kim and Hancock (2015) found that frequent engagement on Facebook increased individuals’ ROB. In addition, there is a significant and negative relationship between social media involvement and optimistic bias (van der Meer et al., 2023).

To date, numerous studies have attempted to evaluate the impact of media exposure and media involvement on cognitive factors (Liu and Li, 2021). Perceived behavioral control is defined as the degree to which people anticipate their competence in performing a certain behavior. In other words, it is an individual’s perception of the difficulty of a specific behavior (Arli et al., 2018). In the current study, perceived prevention of heavy drinking control (PPHDC) refers to Chinese college students’ perception of their ability to decrease their consumption of alcoholic beverages. Numerous studies have explored how frequent exposure to information on social media has an impact on perceived behavioral control (Lwin and Malik, 2014; Bassett-Gunter et al., 2017). A prior study has also stressed that frequent exposure to emergency preparedness videos increased college students’ perceived behavioral control for preventive behaviors (Skurka et al., 2018). Moreover, being exposed to environment-related information is positively associated with individuals’ perceived behavioral control for acquiring environmentally friendly behaviors (Lee, 2011). Many recent studies have explored the effect of social media involvement on individuals’ perceived behavior control. Karimi et al. (2021) confirmed that social media use (media involvement) is positively associated with individuals’ perceived behavioral control for pro-environmental behaviors. Previous research also demonstrated that social media engagement increases individuals’ perceived behavioral control in enrolling in an online university program (Konstantoulaki et al., 2022) and academic self-efficacy (referred to as perceived behavioral control) (Boahene et al., 2019). Thus, the following hypotheses are proposed:

**Hypotheses 1a-c:** EAACSFV has a significant and positive effect on Chinese college students’ (a) WRAC, (b) ROB, and (c) PPHDC.

**Hypotheses 2a-c:** SFVI has a significant and positive effect on Chinese college students’ (a) WRAC, (b) ROB, and (c) PPHDC.

### The mediating roles of ROB and PPHDC

Previous research has demonstrated that exposure to information through social media can induce a positive effect on ROB (Thompson et al., 2002; van der Meer et al., 2023). Furthermore, optimistic bias was negatively related to individuals’ self-protective behaviors during COVID-19 (Chen et al., 2021). Chung’s cross-sectional study (Chung, 2019) demonstrated that exposure to climate-change-related information was positively related to college students’ first-person perception, which further induced individuals’ willingness to share pro-environmental news messages through social media. The existing literature has also explored the relationship between social media involvement and ROB (Kim and Hancock, 2015). For instance, van der Meer et al. (2023) indicated that general media use is negatively associated with optimistic bias (ROB). Moreover, a previous study found that optimistic bias was negatively associated with individuals’ intentions to perform hand hygiene practices (Kim and Niederdeppe, 2013). In other words, increased ROB has increased their willingness to engage in the healthy behavior. Therefore, it is logical that Chinese college students’ ROB would indirectly affect the relationship between EAACSFV and WRAC, as well as the relationship between SFVI and WRAC. Thus, the following hypotheses were proposed:

**Hypothesis 3a:** ROB mediates the relationship between EAACSFV and Chinese college students’ WRAC.

**Hypothesis 3b:** ROB mediates the relationship between SFVI and Chinese college students’ WRAC.

To date, a number of studies have found that greater social media exposure increases individuals’ perceived behavioral control (Lwin and Malik, 2014; Bassett-Gunter et al., 2017; Skurka et al., 2018). For instance, Skurka et al. (2018) revealed the positive association between frequent exposure to emergency preparedness videos and college students’ perceived behavioral control for preventive behaviors. Furthermore, a previous study found that perceived behavioral control for fire prevention increased individuals’ fire preparedness and safety behaviors (Kurata et al., 2023). Similarly, scholars have also found an indirect effect of perceived behavioral control in the relationship between media involvement and individuals’ preventive behaviors. Baker and White (2010) found that social media engagement amplified individuals’ perceived behavioral control of socializing online, which, in turn, raised willingness to share tacit knowledge (Haq et al., 2022). In addition, Fang and Ha (2015) found that social media involvement is positively associated with college students’ perceived political self-efficacy (perceived behavioral control). Ultimately, this induced their online political participation (Yang and DeHart, 2016). Thus, it can be inferred that Chinese college students’ PPHDC could indirectly affect the relationship between EAACSFV and WRAC, as well as the relationship between SFVI and WRAC. Thus, the following hypotheses were proposed:

**Hypothesis 4a:** PPHDC mediates the relationship between EAACSFV and Chinese college students’ WRAC.

**Hypothesis 4b:** PPHDC mediates the relationship between SFVI and Chinese college students’ WRAC.

### The moderating effect of conscientiousness

A previous study has indicated that personality traits are the key factors affecting cognitive factors in the context of online information processing (Power et al., 2017). Moreover, among the different personality traits, conscientiousness strongly predicts health outcomes (e.g., reduction in cigarette smoking and COVID-19 preventative health behaviors) (Hampson et al., 2000; Otterbring and Festila, 2022). Conscientiousness, one of the “big five” personality traits, is the tendency to be hard working, organized, goal directed, and more adjusted to social norms and rules (Otterbring and Festila, 2022). In addition, conscientiousness is defined as being a person who has higher cognitive thinking processes, such as problem-focused coping and less avoidant coping (Wrosch and Scheier, 2003). A handful of studies have also demonstrated that individuals with conscientiousness are associated with a higher willingness to acquire preventive behaviors (Shook et al., 2020; Hu and Lü, 2022). However, to the best of our knowledge, there has been no study investigating the moderating mechanism of how conscientiousness in individuals affects the relationship between exposure to preventive health information and their risk judgment (ROB).

The available evidence has demonstrated that conscientiousness not only directly affects preventative health behaviors, but this may also serve as a moderator of the link between EAACSFV and ROB. For instance, more conscientious individuals tend to estimate the risk of COVID-19 as being more likely to infect themselves than others (ROB) and strongly believe themselves to be part of the high-risk population, who are more vulnerable to COVID-19 than others (Krupić et al., 2021). Moreover, one study indicated that conscientious citizens have an increased willingness to acquire COVID-19-related protection (Moore et al., 2022). One of the possible reasons for this behavior is that they believe they have an increased likelihood of being infected by the disease than others (ROB). Specifically, conscientious citizens show more amplified anxiety and fearful feelings compared to people with other types of personality traits (Bansal and Gefen, 2010). As mentioned above, previous studies have demonstrated that conscientiousness is associated with risk judgment. Therefore, it is logical that this could play a role as a moderating mechanism in the relationship between EAACSFV and Chinese college students’ ROB. Hence, the following hypothesis was proposed:

**Hypothesis 5:** Conscientiousness moderates the relationship between EAACSFV and Chinese college students’ ROB.

**FIGURE 1:**
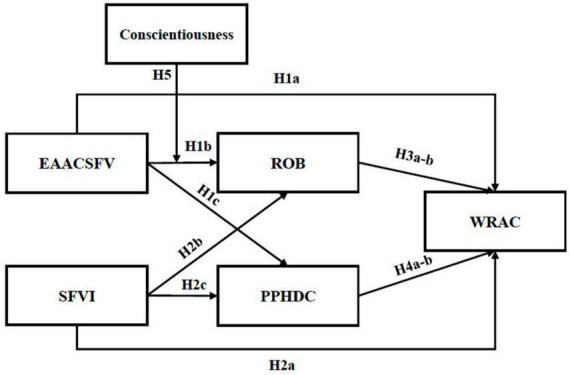
Model of predictors of the willingness to reduce alcohol consumption.

In summary, Figure 1 and shows the theoretical model summarizing the hypotheses.

## Materials and methods

### Questionnaire design

The current study designed 6 measures (EAACSFV, SFVI, ROB, PPHDC, WRAC, and conscientiousness) from prior studies (Dono et al., 2021; Liu and Li, 2021; Wolff, 2021; Steyn and Ndofirepi, 2022). Since each scale has not been applied in the context of China before, three modifications were made to ensure the precision and accuracy of the measures (Broder et al., 2007; Seo et al., 2016; Zhang et al., 2019). Firstly, three Mandarin Chinese-speaking language experts were invited to translate the measures from English to Mandarin Chinese. Later, the researchers compared the original and translated measures to ensure the translation quality (Zhang et al., 2019). Secondly, two experts from the field of media studies, who were not the authors, employed the content and face validity methods to revise and remove items of measurement (Broder et al., 2007) from the current study. Lastly, 10 volunteers were recruited from the Beijing Institute of Graphic Communication, and they were asked to offer suggestions if any item was difficult to read or felt confusing (Seo et al., 2016). Based on the modification methods mentioned above, the questionnaire was carefully revised and uploaded on a Chinese online survey platform, Wenjuanxing. Currently, Wenjuanxing is one of the most used survey design and dissemination platforms in China (Tan et al., 2021). This online survey platform provides a sampling pool of nearly 260 million registered users in China. In addition, the platform has broadly been applied to market surveys, as well as for research purposes (She et al., 2021; Ng et al., 2022). Permission to conduct the current research was reviewed and approved by the Beijing Institute of Graphic Communication Academic Committee (SC20221101). Informed consent was obtained from all participants prior to the start of the survey, with special consideration given to the unique ethical concerns associated with conducting a COVID-19 study. The following criteria were used to determine eligibility for this study: (1) Age 18–24 years, (2) Residence in mainland China during COVID-19, (3) Currently enrolled in undergraduate or graduate programs at universities in China. The participants were recruited from September 1st, 2022, to November 1st, 2022, through the online survey platform. Among the 450 respondents who clicked the link, 434 properly completed the questionnaire.

### Measurement of variables

A five-point Likert scale was applied to all six measurements (EAACSFV, SFVI, ROB, PPHDC, WRAC, and conscientiousness). Respondents rated their agreement ranging from 1 (strongly disagree) to 5 (strongly agree) or from 1 (very rarely) to 5 (very frequently).

### EAACSFV

To measure EAACSFV, the current study modified Liu and Li (2021) exposure to environmental issues information scale by changing the subject from environmental issues to anti-alcohol consumption. It was an index that measured how often participants watch content related to “responsible drinking,” “criminal behavior after alcohol consumption,” and “excessive alcohol consumption leading to a series of health problems” on the following four applications: (a) Douyin, (b) WeChat short videos, (c) Kuaishou, and (d) Watermelon video (*M* = 2.41, SD = 1.04, α = 0.85).

### SFVI

The three-item SFVI index measures how often individuals engage in activities related to “anti-alcohol consumption” issues on short-form video applications. This was adopted from Kalogeropoulos et al. (2017) study. Respondents were asked to indicate their level of agreement with the following questions: “During an average week, in what ways, if any, do you involve related to anti-alcohol consumption issues on short-form video applications?” Respondents answered using a five-point Likert scale for (a) liking contents, (b) commenting on a video, and (c) sharing short-form videos (*M* = 2.03, SD = 1.10, α = 0.85).

### ROB

Reverse optimistic bias measures an individual’s belief that extreme alcohol consumption will result in a higher risk to themselves compared to others. To the best of our knowledge, no study has developed an optimistic bias scale in the Chinese context. Therefore, this study conducted a face validity test of the original measure derived from Khan and Khalid (2023). Following the approach outlined by Zechariah et al. (2021), six panels of experts in the field of health communication were invited (selected from universities in Shanghai). The measure was carefully reviewed with a focus on three items, using an eight-point dichotomous criterion with the options “clear” and “not clear.” The overall design of the optimistic bias scale was found to have poor face validity. Specifically, all expert panels indicated that the “scoring system is not clear and easy to use” and that the “total score of the instrument is not accurately calculated.”

To achieve greater precision in measuring ROB within the Chinese context, we developed an instrument following Lee and Jan (2022) process. First, 12 valid items for the ROB scale related to alcohol consumption were selected from relevant literature sources such as journals and Chinese medical reports. Second, a focus group was convened to confirm the scope, content, purpose, and applicability of the ROB concept to the real-life situations of Chinese college students. For this purpose, five health communication experts (selected from universities in Beijing and Shanghai) were invited to participate in the focus group discussion, which took place on August 20, 2022. During this process, 4 items were removed. Finally, a pilot test was conducted with 40 college students recruited from the Beijing Institute of Graphic Communication. Based on the results of the pilot test, four items were removed due to poor performance. Respondents rated their level of agreement using a five-point Likert scale on the following four questions about long-term alcohol consumption: “I may become more irritable than others,” “It may disrupt my life and work rhythm compared to others,” “It will have a serious effect on my mental state (confusion, poor sleep quality, etc.),” and “Long-term alcohol consumption may lead to more serious health consequences for me than for others (such as high blood pressure and liver damage)” (*M* = 3.11, SD = 1.00, α = 0.71).

### PPHDC

The five items of the PPHDC index were derived from the studies of Young et al. (2007) and Wolfe and Higgins (2008). This study measures Chinese college students’ perceptions of their ability to reduce their consumption of alcoholic beverages. Respondents were asked to rate their confidence in their ability to resist alcohol consumption using four statements: (1) “I am confident that I can resist drinking when I am alone,” (2) “I make sure that I can refuse when I am worried,” (3) “I believe that I have the ability not to drink when I am at a party,” and (4) “I am very sure that I can resist drinking when I am angry” (*M* = 3.40, SD = 1.10, α = 0.85).

### WRAC

The WRAC index was a modified version of Paswan et al. (2015) scale. This scale assessed respondents’ willingness to reduce alcohol consumption in the future. Respondents indicated their level of agreement with the following four statements: (1) “I would like to stop drinking,” (2) “I could accomplish a lot if I could just stop drinking,” (3) “I would like to cancel any event next week where I would have to drink with friends,” and (4) “I have often thought about quitting drinking” (*M* = 3.90, SD = 1.01, α = 0.62).

### Conscientiousness

Conscientiousness was measured using the brief version of the Big Five Inventory (Steyn and Ndofirepi, 2022). The inventory includes 10 items that measure each personality dimension, namely, extroversion, agreeableness, conscientiousness, neuroticism, and openness to experience. However, in this study, the four other personality factors were not included for analysis. This scale uses a five-point Likert scale, and the responses ranged from 1 (never) to 5 (always) (*M* = 3.20, SD = 0.54, α = 0.63).

## Analysis

In this study, the analysis of the accumulated data was carried out using SPSS 22. The software was used in the analysis of descriptive statistics, correlation, Cronbach’s alpha, and hierarchical regression. Hayes’ PROCESS macro for SPSS was used as statistical packages for processing the mediation and moderation analysis. Bootstrapping was then used to obtain bias-corrected 95% confidence intervals to make statistical inferences about specific mediating effects.

## Results

### Descriptive data

In total, 434 valid responses were collected. The demographic characteristics of the survey participants are presented in Table 1. The participants were mostly female (*N* = 268, 61.8%), and undergraduates (*N* = 348, 80.2%) or postgraduates (*N* = 86, 19.8%). The respondents’ ages ranged from 22 to 24 years old (*N* = 247, 56.9%), and they had a monthly family income ranging from CNY 1000 to 6999 (*N* = 224, 51.6%). The bivariate associations among the key variables (independent, dependent, and demographic) are shown in Table 2.

**TABLE 1:** Sample characteristics.

Characteristics	Levels	Frequency	Proportion (%)
Gender	Female	268	61.8%
	Male	166	38.2%
Education Level	Undergraduate	348	80.2%
	Postgraduate	86	19.8%
Age	18–21 years old	187	43.1%
	22–24 years old	247	56.9%
Family Income	RMB 1,000–6999	224	51.6%
	RMB 7000–14,000	141	32.5%
	RMB 14,000–49,999	54	12.4%
	RMB > 50,000	15	3.5%
	Total	434	100%

**TABLE 2:** Correlations among key variables.

Variable	1	2	3	4	5
EAACSFV	–				
SFVI	0.72**	–			
ROB	0.67 **	0.68**	–		
PPHDC	0.13**	0.68	0.61**	–	
WRAC	0.13**	0.28	0.85**	0.45**	–
Conscientiousness	0.87	0.10 *	0.33	0.92	0.16

*p < 0.05;

**p < 0.01. EAACSFV, exposure to anti-alcohol consumption short-form videos; SFVI, short-form video involvement; ROB, reversed optimistic bias; PPHDC, perceived prevention of heavy drinking control; WRAC, willingness to reduce alcohol consumption.

### Hypothesis testing

Hierarchical regression analyses (Huta, 2014) were performed in SPSS (Chambers et al., 2003) to test hypotheses 1a–c and 2a–c. Firstly, demographic factors (gender, education, and income) were entered into the first block as confounding variables. Secondly, EAACSFV and SFVI were entered into the second block as independent variables. Lastly, WRAC, ROB, and PPHDC were added as dependent variables. The effects of EAACSFV on WRAC (β = 0.11, *p* < 0.01), ROB (β = 0.54, *p* < 0.001), and PPHDC (β = 0.12, *p* < 0.05) were all found to be significant. Therefore, H1a-c are fully supported. The effect of SFVI on ROB (β = 0.55, *p* < 0.001) was found to be significant, whereas its effects on WRAC (β = 0.23, *p* > 0.05) and PPHDC (β = 0.58, *p* > 0.05) were not significant. Thus, H2b is supported.

**FIGURE 2:**
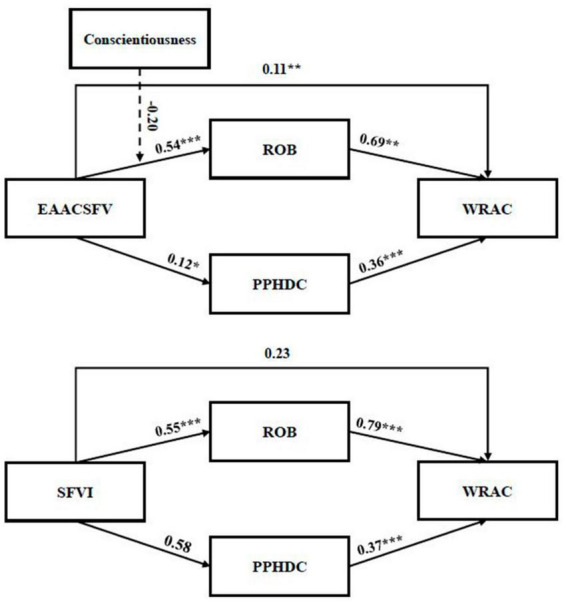
The effects of predictors of willingness to reduce alcohol consumption. *p < 0.05, **p < 0.01, ***p < 0.001.

To examine the mediating effect of EAACSFV and SFVI on Chinese college students’ WRAC (hypotheses 2a–b and 3a–b), Hayes’ PROCESS macro (model 4) (Hayes, 2012) was used in SPSS (Chambers et al., 2003). In addition, the current study applied bootstrap estimates to generate 95% bias-corrected confidence intervals for the observed indirect conditional effects (Bartone and Homish, 2020). Figure 2 indicates the standardized coefficients and significance for each path in the hypothesized model. In the first mediation model, it was demonstrated that ROB positively predicted WRAC (β = 0.69, *p* < 0.01). Meanwhile, EAACSFV was a significant predictor of WRAC (β = 0.26, *p* < 0.05). The indirect effect was significant (β = 0.35, *p* < 0.01, 95% CI [0.17, 0.63]). Therefore, the partial mediating effect of ROB was confirmed. The results of the second mediation model show that ROB positively predicted WRAC (β = 0.79, *p* < 0.001). Meanwhile, SFVI was an insignificant predictor of WRAC (β = 0.15, *p* > 0.05). The indirect effect was significant (β = 0.44, *p* < 0.001, 95% CI [0.20, 0.65]). Therefore, the full mediating effect of ROB was confirmed. In the third mediation model, it was shown that PPHDC positively predicted WRAC (β = 0.36, *p* < 0.001). Meanwhile, EAACSFV was an insignificant predictor of WRAC (β = 0.06, *p* > 0.05). The indirect effect was significant (β = 0.05, *p* < 0.001, 95% CI [0.01, 0.09]). Therefore, the full mediating effect of PPHDC was confirmed. Lastly, the results of the fourth mediation model indicated that PPHDC positively predicted WRAC (β = 0.37, *p* < 0.001). Meanwhile, SFVI was an insignificant predictor of WRAC (β = −0.01, *p* > 0.05). The indirect effect was insignificant (β = 0.02, *p* > 0.05, 95% CI [−0.01, 0.06]). In summary, hypotheses 3a-b and 4a are supported. Table 3 provides a summary of the mediating effects and hypothesis testing results.

**TABLE 3:** Summary of the mediating effect tests.

Indirect effects	B	*P*	LLCI	ULCI
EAACSFV→ ROB → WRAC	0.35	*p* < 0.01	0.17	0.63
SFVI→ ROB → WRAC	0.44	*p* < 0.001	0.20	0.65
EAACSFV→ PPHDC → WRAC	0.05	*p* < 0.001	0.01	0.09
SFVI→ PPHDC → WRAC	0.02	*p* > 0.05	−0.01	0.06

EAACSFV, exposure to anti-alcohol consumption short-form videos; SFVI, short-form video involvement; ROB, reversed optimistic bias; PPHDC, perceived prevention of heavy drinking control; WRAC, willingness to reduce alcohol consumption.

The moderation effect of conscientiousness was analyzed by applying Hayes’ PROCESS macro (model 1) (Hayes, 2012). The results demonstrate the moderating effect of conscientiousness on the relationship between EAACSFV and ROB (EAACSFV x conscientiousness on ROB, β = −0.20, *p* > 0.05, 95% CI [- 0.03, 0.15]). Therefore, H5 is rejected. Figure 2 indicates the standardized coefficients and significance for each path in the hypothesized models of EAACSFV and SFVI for WRAC.

Table 4 provides a summary of the hypotheses, proposed relationships, and hypothesis testing results.

**TABLE 4:** Summary of hypothesis testing results.

Hypothesis	Relationship	Result
H1a	EAACSFV has a significant and positive effect on Chinese college students’ WRAC.	Supported
H1b	EAACSFV has a significant and positive effect on Chinese college students’ ROB.	Supported
H1c	EAACSFV has a significant and positive effect on Chinese college students’ PPHDC.	Supported
H2a	SFVI has a significant and positive effect on Chinese college students’ WRAC.	Not supported
H2b	SFVI has a significant and positive effect on Chinese college students’ ROB.	Supported
H2c	SFVI has a significant and positive effect on Chinese college students’ PPHDC.	Not supported
H3a	ROB mediates the relationship between EAACSFV and Chinese college students’ WRAC.	Supported
H3b	ROB mediates the relationship between SFVI and Chinese college students’ WRAC.	Supported
H4a	PPHDC mediates the relationship between EAACSFV and Chinese college students’ WRAC.	Supported
H4b	PPHDC mediates the relationship between SFVI and Chinese college students’ WRAC.	Not supported
H5	Conscientiousness moderates the relationship between EAACSFV and Chinese college students’ ROB.	Not supported

EAACSFV, exposure to anti-alcohol consumption short-form videos; SFVI, short-form video involvement; ROB, reversed optimistic bias; PPHDC, perceived prevention of heavy drinking control; WRAC, willingness to reduce alcohol consumption.

## Discussion

Guided by the theory of optimistic bias, the current study investigated the relationship between the short-form video media effects (exposure to anti-alcohol consumption short-form videos and short-form video involvement) and willingness to reduce alcohol consumption among Chinese college students (18–24 years old). Moreover, the main objective with this research was to examine the mediating mechanism of reversed optimistic bias and perceived prevention of heavy drinking control in this relationship. Lastly, the role of conscientiousness as a moderator was also thoroughly investigated.

A cross-sectional study was conducted on a sample of Chinese college students. The findings demonstrate that H1a-c are fully supported. In terms of the direct effect, exposure to anti-alcohol consumption short-form videos was a significant predictor of willingness to reduce alcohol consumption among Chinese college students, which is in line with previous findings (Knobloch-Westerwick and Sarge, 2015; Gehrau et al., 2021). For instance, Gehrau et al. (2021) found that the greater the exposure to health information, the greater the intention to get vaccinated against COVID-19. Furthermore, the current study confirmed a positive relationship between Chinese college students’ exposure to anti-alcohol consumption short-form videos and reversed optimistic bias. This finding aligns with those of a previous study that demonstrated a positive association between exposure to HIV prevention videos and reduced perceptions of invulnerability (which is also known as reversed optimistic bias) among college students when compared to their peers (Thompson et al., 2002). Finally, the current study confirmed a strong and positive effect of Chinese college students’ exposure to anti-alcohol consumption short-form videos on their perceived prevention of heavy drinking control. This finding supports the previous evidence that frequent exposure to emergency preparedness videos increases college students’ perceived behavioral control for preventive behaviors (Skurka et al., 2018).

In terms of the direct effects of short-form video involvement, such factor did not predict Chinese college students’ willingness to reduce alcohol consumption, which is contrary to the findings of prior studies (Cao et al., 2017; Zhou and Krishnan, 2019). This can be explained through individuals realizing that a piece of information is considered to be from a low-credibility source, which results in a lower bandwagon cue (Li et al., 2020). In other words, if individuals feel less social pressure and are less attracted to such information, it will further reduce their likelihood of sharing information on social media. This phenomenon may be explained by the significant number of health-related short-form videos that are not verified by health authorities and are created by non-experts in health (Reem, 2022). As a result, this may decrease the willingness of Chinese students to rely on and share such information (willingness to reduce alcohol consumption). Moreover, the current study confirmed that short-form video involvement is a significant predictor of Chinese college students’ reversed optimistic bias, which is aligns with prior studies that showed that involvement in social media (e.g., Facebook) increases individuals’ reversed optimistic bias (Kim and Hancock, 2015; van der Meer et al., 2023). Contrary to expectations, short-form video involvement did not predict Chinese college students’ perceived prevention of heavy drinking control, which is in contrast to the findings of previous studies (Boahene et al., 2019; Karimi et al., 2021; Konstantoulaki et al., 2022). An explanation could be that short-form video involvement is overshadowed by other predictors. For example, Williams and Hine (2002) argued that parental behavior, such as the father’s alcohol consumption, is a strong predictor which increases adult children’ perceived behavior control.

In regard to mediating effects, the findings of H3a-b testing demonstrate that reversed optimistic bias has a mediating effect on exposure to anti-alcohol consumption short-form videos and short-form video involvement on willingness to reduce alcohol consumption among Chinese college students. These findings are in line with previous studies that indicate that exposure to HIV prevention videos amplified individual’s reversed optimistic bias (Thompson et al., 2002), which in turn, had a positive effect on food stockpiling behaviors (Van der Eijk et al., 2013). Moreover, the frequency of social media engagement increased individuals’ reversed optimistic bias (Kim and Hancock, 2015). Eventually, this induced individuals’ interest in participating in COVID-19 preventive behaviors (Carvalho et al., 2020). Another finding indicates that perceived prevention of heavy drinking control mediates the relationship between exposure to anti-alcohol consumption short-form videos and Chinese college students’ willingness to reduce alcohol consumption. In accordance with the present results, prior studies have indicated that exposure to emergency preparedness information increases individuals’ acquisition of preventive behaviors via perceived behavioral control (Skurka et al., 2018). One unanticipated finding was that perceived prevention of heavy drinking control did not mediate the relationship between short-form video involvement and Chinese college students’ willingness to reduce alcohol consumption, which is contrary to previous studies’ findings (Fang and Ha, 2015; Yang and DeHart, 2016). This inconsistency may be due to the serial mediating mechanism of digital health literacy and perceived prevention of heavy drinking control in the relationship between short-form video involvement and willingness to reduce alcohol consumption. For instance, Atique et al. (2016) indicated that social media involvement increases individuals’ level of digital health literacy. Furthermore, self-efficacy (perceived behavior control) shows a mediating effect through digital health literacy on individuals’ adoption of healthy habits. The results of the current study indicate that exposure to anti-alcohol consumption short-form videos plays an important role in increasing willingness to reduce alcohol consumption, reversed optimistic bias, and perceived prevention of heavy drinking control, whereas short-form video involvement demonstrates both an insignificant direct and mediating effect on willingness to reduce alcohol consumption via perceived prevention of heavy drinking control. Thus, this study encourages future research to further clarify the potential effects of the relationship between perceived prevention of heavy drinking control and willingness to reduce alcohol consumption.

In terms of moderating effects, Chinese college students’ conscientiousness did not show a significant effect on the relationship between exposure to anti-alcohol consumption short-form videos and reversed optimistic bias. This inconsistency with previous findings may be attributed to self-affirmation as one of the potential strong moderators in this relationship. For instance, a previous study showed that individuals who demonstrate higher self-affirmation show less defensive reactions when exposed to threatening health-related messages (reversed optimistic bias) (Armitage et al., 2011). A related study has also claimed that individuals who show higher self-affirmation have reduced underestimation of the likelihood of experiencing negative events (reversed optimistic bias) (Pauketat et al., 2016). In light of this, the current study encourages future studies to further investigate the moderating mechanism of self-affirmation.

In terms of theoretical contributions, the current study has enriched our understanding in several ways. First, in the absence of theoretical support related to psychological biases, such as optimistic bias, there has been a limited amount of research examining college students’ alcohol consumption. This study addresses this important gap and provides empirical evidence for the applicability of the theory in the Chinese context. Second, the current study has extended the theoretical framework by providing a deeper understanding of how an inverted optimistic bias influences Chinese college students’ willingness to reduce alcohol consumption under the influence of short-form video media effects. Third, this study supports the idea that both anti-alcohol short-form videos and short-form video engagement increase Chinese college students’ reversed optimistic bias, which subsequently increases their willingness to reduce alcohol consumption. These results are consistent with previous findings (Thompson et al., 2002; Kim and Niederdeppe, 2013; Kim and Hancock, 2015; Chen et al., 2021; van der Meer et al., 2023). Therefore, future studies can examine college students from different regions and countries to compare their risk perception and decision-making from the perspective of reversed optimistic bias.

The current study has several practical implications. Firstly, the current study suggests that in order to create credible alcohol consumption warning videos, the content of short-form videos should be verified and endorsed by health departments or health experts. For example, Mutti-Packer et al. (2017) found that message credibility significantly moderated the mechanism in the relationship between tobacco health risk warnings and the perceived effectiveness of the warnings. In other words, the more credible the message, the more effective the prevention. Secondly, the majority of health-related short-form videos are created by doctors within the context of China. Additionally, these videos have successfully captured the attention of Chinese users and have contributed to the expansion of their health literacy (Sun et al., 2023). Therefore, it is recommended that more physicians create content that promotes the prevention of excessive alcohol consumption. Finally, a previous study emphasized that perceived support is one of the most important predictors with a positive impact on reduction of alcohol consumption (Mavandadi et al., 2015; Paswan et al., 2015). Therefore, Chinese faculty members, family, and friends are encouraged to provide support to college students struggling with alcohol consumption.

## Limitations and conclusion

There are several limitations that should be noted. First, the current study was conducted using a cross-sectional design. Therefore, it could not fully establish causal effects. To enable more comprehensive investigations, future researchers are encouraged to carry out longitudinal or experimental studies. Second, this study operationalized reversed optimistic bias using four items which were developed based on Lee and Jan’s (Lee and Jan, 2022) process. Undoubtedly, the limited number of items makes it difficult to accurately measure Chinese students’ underestimation of the negative effects of alcohol consumption. Thus, future studies should apply more precise measurement tools. Finally, the gender distribution of respondents in the current study was predominantly female, which is not consistent with the national gender distribution in China. The use of representative sampling methods and a larger number of Chinese students is highly recommended.

The main goal of the current study was to explore the complex relationship between the short-form video media effects (exposure to anti-alcohol consumption short-form videos and short-form video involvement) and willingness to reduce alcohol consumption among Chinese college students (18–24 years old). Moreover, this is one of the few studies to discover the mediating effects of a cognitive factor (perceived prevention of heavy drinking control) and psychological bias (reversed optimistic bias), and the moderating mechanisms of conscientiousness in this relationship. This study confirms that exposure to anti-alcohol consumption short-form videos plays an important role in increasing willingness to reduce alcohol consumption via reversed optimistic bias, and perceived prevention of heavy drinking control. Additionally, short-form video involvement shows both insignificant direct and mediating effects on willingness to reduce alcohol consumption via perceived prevention of heavy drinking control. Lastly, conscientiousness did not moderate the relationship between exposure to anti-alcohol consumption short-form videos and reversed optimistic bias. The findings of this study contribute to health psychology studies in the following aspects. Firstly, this is the first study that examined the theory of optimistic bias in explaining college students’ willingness to reduce alcohol consumption within the context of China. Secondly, this study fills the gap in the understanding of two different short-form video effects (exposure to anti-alcohol consumption short-form videos and short-form video involvement) on Chinese college students’ willingness to reduce alcohol consumption. Future studies are encouraged to further investigate the moderating mechanism of self-affirmation in such a relationship.

## Data availability statement

The original contributions presented in the study are included in the article/supplementary material, further inquiries can be directed to the corresponding author.

## Ethics statement

The studies involving humans were approved by the Beijing Institute of Graphic Communication Academic Committee (SC20221101). The studies were conducted in accordance with the local legislation and institutional requirements. The participants provided their written informed consent to participate in this study. Written informed consent was obtained from the individual(s) for the publication of any potentially identifiable images or data included in this article.

## Author contributions

DC and YM: conceptualizing the research idea and writing–original draft, revising the manuscript, processing data and visualizing, and data-collecting and providing revised advice. Both authors contributed to the article and approved the submitted version.
